# Costs of a Motivational Enhancement Therapy Coupled with Cognitive Behavioral Therapy versus Brief Advice for Pregnant Substance Users

**DOI:** 10.1371/journal.pone.0095264

**Published:** 2014-04-23

**Authors:** Xiao Xu, Kimberly A. Yonkers, Jennifer P. Ruger

**Affiliations:** 1 Department of Obstetrics, Gynecology and Reproductive Sciences, School of Medicine, Yale University, New Haven, Connecticut, United States of America; 2 Department of Psychiatry and Department of Obstetrics, Gynecology and Reproductive Sciences, Yale University, School of Medicine, Yale University, New Haven, Connecticut, United States of America; 3 Yale University, Department of Health Policy and Management, New Haven, CT Department of Public Health, School of Medicine, Yale University, New Haven, Connecticut, United States of America; Groningen Research Institute of Pharmacy, Netherlands

## Abstract

**Objectives:**

To determine and compare costs of a nurse-administered behavioral intervention for pregnant substance users that integrated motivational enhancement therapy with cognitive behavioral therapy (MET-CBT) to brief advice (BA) administered by an obstetrical provider. Both interventions were provided concurrent with prenatal care.

**Methods:**

We conducted a micro-costing study that prospectively collected detailed resource utilization and unit cost data for each of the two intervention arms (MET-CBT and BA) within the context of a randomized controlled trial. A three-step approach for identifying, measuring and valuing resource utilization was used. All cost estimates were inflation adjusted to 2011 U.S. dollars.

**Results:**

A total of 82 participants received the MET-CBT intervention and 86 participants received BA. From the societal perspective, the total cost (including participants’ time cost) of the MET-CBT intervention was $120,483 or $1,469 per participant. In contrast, the total cost of the BA intervention was $27,199 or $316 per participant. Personnel costs (nurse therapists and obstetric providers) for delivering the intervention sessions and supervising the program composed the largest share of the MET-CBT intervention costs. Program set up costs, especially intervention material design and training costs, also contributed substantially to the overall cost.

**Conclusions:**

Implementation of an MET-CBT program to promote drug abstinence in pregnant women is associated with modest costs. Future cost effectiveness and cost benefit analyses integrating costs with outcomes and benefits data will enable a more comprehensive understanding of the intervention in improving the care of substance abusing pregnant women.

## Introduction

Substance use during pregnancy is a major public health problem in the United States. In the 2011 National Survey on Drug Use and Health, 9.4% of pregnant women aged 15 to 44 reported current alcohol use, with 2.6% and 0.4% reporting binge drinking and heavy drinking, respectively [1]. The survey data also showed that 5.0% of pregnant women used illicit drugs in the month preceding the survey with marijuana being the most commonly used illicit drug, while 17.6% of pregnant women reported cigarette use in the past month [1]. Prenatal substance use carries adverse health consequences for both the mother and the newborn. For instance, in-utero exposure to alcohol and illicit drugs is associated with poor fetal growth, preterm birth, low birth weight, behavioral problems, and cognitive and developmental disabilities [2], [3]. Illicit drug use during pregnancy also increases maternal risk for HIV infection, which in turn increases the risk for perinatal transmission of HIV and adverse birth outcomes [4], [5], [6], [7].

Because of these complications and associated short- and long-term maternal and neonatal morbidities, financial consequences of substance use during pregnancy can be substantial. Compared to unexposed infants, drug-exposed infants have longer average lengths of stay and higher average rates of admission to neonatal intensive care units [8], [9]. Nationwide the overall medical care costs for drug exposed infants were estimated to be $605 million in 2002 [10].

Therefore, substance use and HIV prevention programs targeting pregnant women have the potential to be cost-beneficial. However, while some interventions have demonstrated significant clinical benefit (e.g., the Kaiser Permanente program of universal substance use risk screening and education for pregnant women and ongoing professional consultation and training for obstetric providers [11]), few economic evaluations of such programs have been conducted. A review of the literature identified only nine studies examining the cost consequences of drug abuse treatment programs for pregnant women [12]. Most of these studies relied on data that were more than a decade old and all were based on non-randomized study designs and hence were subject to bias and confounding effects [12].

In addition, *none* of the existing studies used micro-costing methodology that involves the collection of detailed data on input resources utilized and the value of those resources [13]. Previous research has demonstrated that using bottom-up micro-costing methods to measure important cost components helps improve the validity and reliability of total cost estimates for hospital services [14] and for diagnostic or treatment interventions where costs are evolving [15]. The U.S. Panel on Cost Effectiveness in Health and Medicine also recommended micro-costing as the preferred approach when the alternative gross-costing estimation was expected to cause bias [13]. Substance abuse treatment interventions for pregnant women are typically new management strategies without established aggregative cost estimates and often require direct measurement of costs. Hence employment of a micro-costing method is particularly important as it has been shown effective in estimating the cost of new technologies or interventions, and it helps improve precision [16], [17].

The purpose of this study was to conduct a micro-costing analysis within the context of a randomized controlled trial (RCT) to determine the costs of a behavioral intervention, i.e., motivational enhancement therapy (MET) integrated with cognitive behavioral therapy (CBT) administered by a nurse, as compared to brief advice (BA) from an obstetrical provider, for pregnant substance users. Findings from this study will facilitate subsequent assessment of the cost effectiveness of these treatment interventions in pregnant women and inform clinical and policy decision making. In the current climate of escalating health care costs, such information is instrumental for ensuring efficient resource allocation and sustainable health care programs.

## Materials and Methods

### Ethics Statement

The study was reviewed and approved by Yale University and Bridgeport Hospital Human Investigation Committees. We obtained written informed consent from all study participants.

### Recruitment, Design, and Sample of the Randomized Controlled Trial

We conducted an economic analysis alongside an RCT that compared the efficacy of motivational enhancement therapy coupled with cognitive behavioral therapy (MET-CBT) to brief advice (BA) for treatment of substance use in pregnancy, reducing potentially harmful sexual behaviors, and improving birth outcomes. To be eligible for the study, women had to be 16 years of age or older, fluent in English or Spanish, had not yet completed 28 estimated weeks of pregnancy at screening, planning to deliver at a collaborating hospital and using alcohol or an illicit drug (other than opiates) during the 28 days prior to screening or scored at least a “3” on the modified TWEAK (Tolerance, Worried, Eye-openers, Amnesia, K[C] Cut Down) screening test [18], [19], [20]. Women were ineligible if they were already receiving substance use treatment, endorsed nicotine or opiates as their only substance, planned to relocate, required inpatient general medical/psychiatric treatment, or were an imminent danger to themselves or fetus. The study excluded women with nicotine as their only substance of abuse because the Yale New Haven Health System already had standard treatment protocols and program on site for them. A certificate of confidentiality was received from the National Institute of Drug Abuse for the purposes of screening and treating this group of at-risk patients.

Study participants were recruited from two hospital-based reproductive health clinics in New Haven and Bridgeport, CT, between June 2006 and July 2010. A total of 183 pregnant women were enrolled in the study and randomized. Research assessments were conducted before the intervention (i.e., baseline assessment), at endpoint (as close to delivery date as possible), as well as at 3 months, 12 months and 24 months after delivery. Fifteen participants did not complete any research assessment or intervention session, because they miscarried, relocated, withdrew from the study, or were out of contact. Therefore, our analysis was based on the remaining 168 participants (n = 82 in the MET-CBT arm and n = 86 in the BA arm).

Details about the design of the RCT, including study procedures, power analysis, and the CONSORT diagram, have been reported elsewhere [21]. The RCT was registered in ClinicalTrials.gov (study name: Therapeutic Substance Abuse Treatment in Pregnancy-1 (PRIDE-P), study identifier: NCT00227903).

### Intervention Conditions

#### Motivational Enhancement Therapy coupled with Cognitive Behavioral Therapy (MET-CBT)

MET-CBT was an individual based therapy combining MET and CBT [22]. It was formatted into six topics that could be delivered in conjunction with prenatal and immediate postnatal care visits: motivational enhancement, functional analysis (non-drug activities and triggers/patterns), safe sexual behavior, communication skills, relapse prevention, and problem-solving skills. MET-CBT sessions were conducted by nurse therapists and delivered after regular prenatal and postnatal appointments. Each session lasted about 30 minutes, and participants completed an average of 5.2 sessions. The nurse therapists could adapt and repeat topics as appropriate to the needs of the participants.

#### Brief Advice (BA)

BA was a short manualized discussion (approximately 1 minute) at the regular prenatal and postnatal visits about the risks of substance use, importance of abstinence, and benefit of seeking drug and alcohol treatment outside of the prenatal setting [22]. The discussion was delivered by the obstetric provider and was repeated at each prenatal and immediate postnatal visit. Obstetric providers included obstetrician-gynecologists (mostly resident physicians and fellows), certified nurse-midwives, nurses, and physician assistants. Participants received an average of 7.2 sessions.

### Micro-costing Methodology

We conducted cost analysis of each of the two intervention arms (MET-CBT and BA) from both a health care system’s perspective and from a more comprehensive societal perspective. The costs assessed in this study were consistent with recommendations by the U.S. Panel on Cost Effectiveness in Health and Medicine to use standardized techniques [13] and methodologies developed and employed in previous studies by members of our research team [23], [24], [25]. The micro-costing method entailed a three-step approach that identified, estimated and valued resource utilization for each intervention arm.

#### Identifying resources used

In step 1, it is important to thoroughly delineate all inputs that were used by the intervention so as to account for the cost of each component. This first step involves the identification of resources used in the “production process” of the intervention. Each “work step” in the production process is then described in conjunction with the relevant cost components. The costs for delivering the MET-CBT and BA interventions were partitioned into four categories: (1) set up costs, which included costs incurred during the initiation of the program such as personnel time spent interviewing and hiring clinical staff, designing intervention and training materials, training providers, and outreaching, as well as non-personnel costs associated with setting up the program administrative office, outreach, and training activities; (2) time-dependent program costs, which included costs that were independent of the number of participants in the program but incurred for as long as the program operated such as personnel time spent on program supervision and costs associated with the use of office space, utilities, equipment, furniture, office supplies and other miscellaneous supplies; (3) variable program costs, which varied with the number of participants and included personnel time spent screening participants for eligibility for the intervention, scheduling participants, preparing and delivering the sessions, and collecting urine and breath sample, as well as costs associated with the use of exam room at clinic, urine and breath test supplies, intervention materials, and transportation; and 4) societal costs, which included participants’ time in intervention sessions and travel. Both set up costs and time-dependent program costs are fixed costs that do not vary with the number of participants. Set-up costs are one-time costs incurred when initiating the program, while time-dependent program costs are on-going while the program is in operation.

#### Measuring resource use

The next step in micro-costing is to measure the inputs to the intervention. To enhance the reliability and validity of our cost data, we drew on several mechanisms to maximize our ability to measure inputs consumed prospectively (as part of the RCT), rather than trying to reconstruct them retrospectively. We audio-taped both MET-CBT and BA intervention sessions (with participants’ permission), which enabled accurate measurement of session duration. Data collection forms were designed and completed prospectively to track the exact topic(s) delivered at each intervention session, therapists and providers involved, distribution of intervention materials, and collection of biochemical assessments (i.e., urine and breath tests). We performed detailed record analysis (including comprehensive transactions records from the business operations database, as well as invoices, billing records and expense reports maintained by the research team) to determine quantity of materials and services consumed during the interventions. Size of all relevant office space and exam rooms were measured based on actual square footage. For participants’ transportation, we used Google Maps (http://maps.google.com/maps) to estimate their round-trip public transit time (base case analysis) and driving time and distance (sensitivity analysis) between the zip code of their home address and the zip code of the clinic where participants received the intervention. Because most intervention sessions were provided at the same visit when participants attended the clinic for prenatal and postnatal care, we only included transportation costs when the participants had intervention sessions outside routine clinic visit. These efforts were supplemented by periodic interviews with program manager and other key personnel regarding additional resource utilizations and personnel involvement in delivering the interventions (e.g., trainers’ time spent on training, and clinical personnel’s time spent on clinical supervision).

#### Valuing resources

Once resource utilization was measured, the quantity of each type of resource consumed was multiplied by unit costs, and the results were summed to obtain total component-specific costs and overall costs. Total and component-specific costs were then divided by the number of participants to determine the average costs per participant. Measurement units for each type of resource utilized in the interventions and our method of valuation were summarized in Table 1. Several approaches were used to estimate the unit costs. For clinical personnel time (e.g., registered nurse, attending physician, resident physician, certified nurse midwives, physician assistant, etc.), we used anonymous salary and fringe benefit information (or average salary and fringe benefit corresponding to the job position at the employee’s institution when actual salary data were not available) to estimate hourly wage rate. For intervention participants, we used monthly earning data in New Haven (matched to each participant based on gender and education) and average number of work hours per month in Connecticut to calculate their hourly wage rate [26], [27]. Annual rent per square foot was based on the rate at the reproductive health clinic in New Haven, CT for clinical space and the actual rent per square foot for the program’s administrative office. In both cases, the rent covered the costs of utilities such as water and electricity. Round-trip transportation to and from the clinic was based on the actual fare of public transportation (in base case analysis) and the standard business travel mileage rates published by the Internal Revenue Service (IRS) (in sensitivity analysis) [28], [29], [30], [31], [32]. The monthly costs of telephone rental, copier rental, and information technology (IT) support were based on the actual rate for the program’s administrative office. The unit costs of all other supplies, equipment, and furniture were based on their actual purchased cost.

**Table 1:** Resource utilization categories and cost measurement.

Resource Utilization Category	Measurable Units	Resource Valuation
Personnel time (e.g., therapist, supervisor, clinic staff)	Hours	Hourly wage rate (includes fringe benefit)
Facility and utilities (e.g., office space, exam room)	Square footage	Annual rent per square foot
Training and intervention materials (e.g., manuals, brochures, information sheets, booklets)	Item	Purchased cost
Urine and breath test	Item	Purchased cost
Equipment (e.g., computers, printers)	Item	Purchased cost
Furniture (e.g., file cabinet, chairs)	Item	Purchased cost
Office supplies (e.g., paper, folders, binders, staplers)	Item	Purchased cost
Refreshments	Item	Purchased cost
Information technology (IT) support	Month	Monthly service expense per person
Telephone rental	Month	Monthly rental expense per person
Transportation	Trip	Round trip fare of public transportation
Patient/participant time	Hours	Hourly wage rate

### Research Costs

As the goal was to estimate the costs necessary for reproducing the interventions in a non-research setting, activities associated with the research objectives of the study were excluded. For example, time spent by research staff on completing survey intake and follow up, data entry and analysis were excluded. Personnel time (e.g., nurse therapists) in preparing and delivering the interventions was distinguished from their time in research activities. Other resources used for research purposes only (e.g., research assistants’ office space, laptop for data entry, and office supplies for research activities) were also excluded from the cost analysis.

### Sensitivity Analyses

We used non-parametric bootstrapping approach to calculate the 95% confidence intervals (CIs) for the per participant total cost estimate for each group. In addition both one-way and two-way sensitivity analyses were conducted to analyze the variation in total costs according to uncertainties in key cost categories. In one-way sensitivity analyses, we assessed changes in per participant total costs in each of the following scenarios: 1) increasing the number of intervention participants by 25%; 2) excluding training costs (i.e., assume existing clinical staff were already capable of running the intervention and delivering the therapy); 3) assuming participants drove to the clinic instead of taking buses (account for productivity loss associated with the time involved in travel as well as the expense of transportation); 4) replacing nurse MET-CBT therapists with social workers; and 5) assuming the program was implemented in a non-academic setting (i.e., replace the wage rate of resident physicians and fellows who delivered intervention sessions with wage rate of attending physicians). In two-way sensitivity analyses, we evaluated the impact on per participant total costs when 1) simultaneously increasing the number of intervention participants by 25% and excluding training costs, and 2) simultaneously increasing the number of intervention participants by 25% and replacing nurse MET-CBT therapists with social workers.

## Results

### Total Program Costs and Costs per Participant

Table 2 shows the total costs and per participant costs for the interventions. All cost estimates were inflation adjusted to 2011 U.S. dollars using the all item (for participant time cost) and medical component (for costs of all other resources) of the consumer price index published by the U.S. Bureau of Labor Statistics [33]. From the health care system’s perspective (i.e., not accounting for participants’ time cost), the total cost of the MET-CBT intervention for the 82 participants was $113,273 or $1,381 per participant (95% CI: $1,341–$1,419). In contrast, the total cost of the BA intervention for the 86 participants was $24,952 or $290 per participant (95% CI: $279–$300). From the societal perspective, the total costs (including participants’ time cost) for the two interventions were $120,483 and $27,199, respectively, resulting in a per participant cost of $1,469 (95% CI: $1,422–$1,514) and $316 (95% CI: $302–$330), respectively.

**Table 2:** Estimated total and per participant costs of Brief Advice (BA) and Motivational Enhancement Therapy with Cognitive Behavioral Therapy (MET-CBT).^a^

Cost Category	Brief Advice (n = 86)		Motivational Enhancement Therapy with Cognitive Behavioral Therapy (n = 82)	
	Total Costs	Costs perParticipant	Total Costs	Costs per Participant
**Set Up Costs** b				
Initial clinic integration^c^	$2,198.17	$25.56	$5,731.79	$69.90
Intervention material design	$371.94	$4.32	$19,619.89	$239.27
Set-up program administrative office	$127.31	$1.48	$127.31	$1.55
Outreach activities	$1,486.07	$17.28	$1,486.07	$18.12
Refreshments	$83.29	$0.97	$83.29	$1.02
Training costs	$2,185.29	$25.41	$22,994.26	$280.42
Transportation - training participants^d^	$20.83	$0.24	$253.94	$3.10
***Subtotal***	***$6,472.91***	***$75.27***	***$50,296.55***	***$613.37***
**Time-Dependent Program Costs** e				
Continued clinic integration^f^	$3,892.68	$45.26	$21,803.87	$265.90
Facility and utilities costs^g^	$1,243.34	$14.46	$7,361.12	$89.77
Other utilities				
Information technology (IT) support	$74.08	$0.86	$1,025.78	$12.51
Phone rental	$64.58	$0.75	$527.36	$6.43
Copier rental	$885.97	$10.30	$885.97	$10.80
Equipment				
Computers	$149.73	$1.74	$1,680.32	$20.49
Printers	$205.95	$2.39	$205.95	$2.51
Shredder	$53.57	$0.62	$53.57	$0.65
Furniture	$215.58	$2.51	$215.58	$2.63
Office supplies				
Toner cartridges	$464.16	$5.40	$464.16	$5.66
Paper	$160.44	$1.87	$160.44	$1.96
Folders/binders	$45.82	$0.53	$45.82	$0.56
Binder clips/paper clips	$3.22	$0.04	$3.22	$0.04
Pens/markers	$17.74	$0.21	$17.74	$0.22
Post it note-pads/flags	$22.56	$0.26	$22.56	$0.28
Calculators	$2.74	$0.03	$2.74	$0.03
Calendar/planner/organizer	$22.44	$0.26	$22.44	$0.27
Stapler/staples/staple remover	$16.64	$0.19	$16.64	$0.20
Clipboards	$11.09	$0.13	$11.09	$0.14
Correction tape/film/liquid	$5.19	$0.06	$5.19	$0.06
Envelops/letterhead	$43.41	$0.50	$43.41	$0.53
Index card/other special paper products	$2.72	$0.03	$2.72	$0.03
Labels	$18.83	$0.22	$18.83	$0.23
Legal pads	$4.21	$0.05	$4.21	$0.05
Protective sheets	$2.37	$0.03	$2.37	$0.03
Message stamps	$1.40	$0.02	$1.40	$0.02
Paper trimmers	$1.91	$0.02	$1.91	$0.02
Scissors	$1.91	$0.02	$1.91	$0.02
Shelf savers baskets	$10.24	$0.12	$10.24	$0.12
Wrist-rests	$6.50	$0.08	$6.50	$0.08
Miscellaneous other supplies				
Cleaning supplies	$2.66	$0.03	$2.66	$0.03
Printer fuser/transfer kit	$18.35	$0.21	$18.35	$0.22
Office/cabinet keys	$3.99	$0.05	$3.99	$0.05
Wastebasket	$0.87	$0.01	$0.87	$0.01
Trash bags	$6.19	$0.07	$6.19	$0.08
Other supplies (packaging tapesshredder lubricant, etc.)	$5.94	$0.07	$5.94	$0.07
***Subtotal***	***$7,689.00***	***$89.41***	***$34,663.05***	***$422.72***
**Variable Program Costs** h				
Personnel time				
Eligibility screening	$1,610.38	$18.73	$1,536.47	$18.74
Scheduling	$57.80	$0.67	$56.14	$0.68
Provider time	$554.34	$6.45	$20,854.65	$254.32
Collection of urine/breathsample and conduction of the tests	$910.56	$10.59	$599.06	$7.31
Testing supplies				
Urine test	$4,523.72	$52.60	$3,028.79	$36.94
Breath test	$472.28	$5.49	$289.77	$3.53
Exam room and utilities	$20.42	$0.24	$354.11	$4.32
Intervention materials				
Brochure	$989.80	$11.51	$640.47	$7.81
Referral information sheets	$1,172.61	$13.64	$505.94	$6.17
Patient worksheets	$0.00	$0.00	$21.35	$0.26
Provider information sheets	$16.24	$0.19	$0.00	$0.00
Handout folders	$103.17	$1.20	$98.37	$1.20
Medical charts	$222.46	$2.59	$212.12	$2.59
March of Dimes PregnancyBaby books	$46.04	$0.54	$42.24	$0.52
Transportation-interventionparticipants	$90.36	$1.05	$74.39	$0.91
***Subtotal***	***$10,790.19***	***$125.47***	***$28,313.87***	***$345.29***
**Societal Cost**				
Intervention participants	$1,804.40	$20.98	$4,503.45	$54.92
Training participants^c^	$442.61	$5.15	$2,706.38	$33.00
***Subtotal***	***$2,247.01***	***$26.13***	***$7,209.83***	***$87.92***
	**Total**	**Per Participant Total Cost (95% confidence interval)**	**Total**	**Per Participant Total Cost (95% confidence interval)**
**Total (health care system** **perspective)**	**$24,952.11**	**$290.14 ($279.22 - $300.21)**	**$113,273.48**	**$1,381.38 ($1,341.31 - $1,419.21)**
**Total (societal perspective)**	**$27,199.12**	**$316.27 ($301.84 - $330.36)**	**$120,483.31**	**$1,469.31 ($1,421.70 - $1,514.06)**

N/A = Not Applicable (the cost is the same across participants).

a For all cost categories reported here, only clinical costs were included (research costs were excluded).

b Set up costs are costs incurred during the initiation of the program and include personnel time spent interviewing and hiring clinical staff, meetings, designing intervention and training materials, training providers, and outreaching, as well as non-personnel costs associated with setting up the program administrative office, outreach, and training activities.

c Activities related to initial clinic integration include hiring clinical personnel and meetings with the staff and administrators at clinic during the initial year of the program to introduce the program, incorporate the program into existing clinic flow, and secure space availability for the intervention sessions.

d During the initial program set up period, training participants were recruited to help providers gain experience with the actual intervention and pilot test the process of delivering the intervention at the clinic.

e Time-dependent program costs are costs that are independent of the number of participants in the program but incurred for as long as the program operates, and include personnel time spent on meetings and program supervision, as well as costs associated with the use of office space, utilities, equipment, furniture, office supplies and other miscellaneous supplies.

f Activities related to continued clinic integration include updating clinical documents (e.g., referral information sheet), meetings and other interactions with the clinic to maintain engagement of clinic staff and providers, and resolving any issues about running the program in the clinical setting.

g These are costs associated with the facility and utilities of the program administrative office where the therapists and supervisors were housed.

h Variable program costs are costs that vary with the number of participants and include personnel time spent screening participants for eligibility into the program, scheduling participants, preparing and delivering the sessions, and collecting urine and breath sample, as well as costs associated with the use of exam room at clinic, urine and breath test supplies, and intervention materials.

### Cost Breakdown

**Figure 1:**
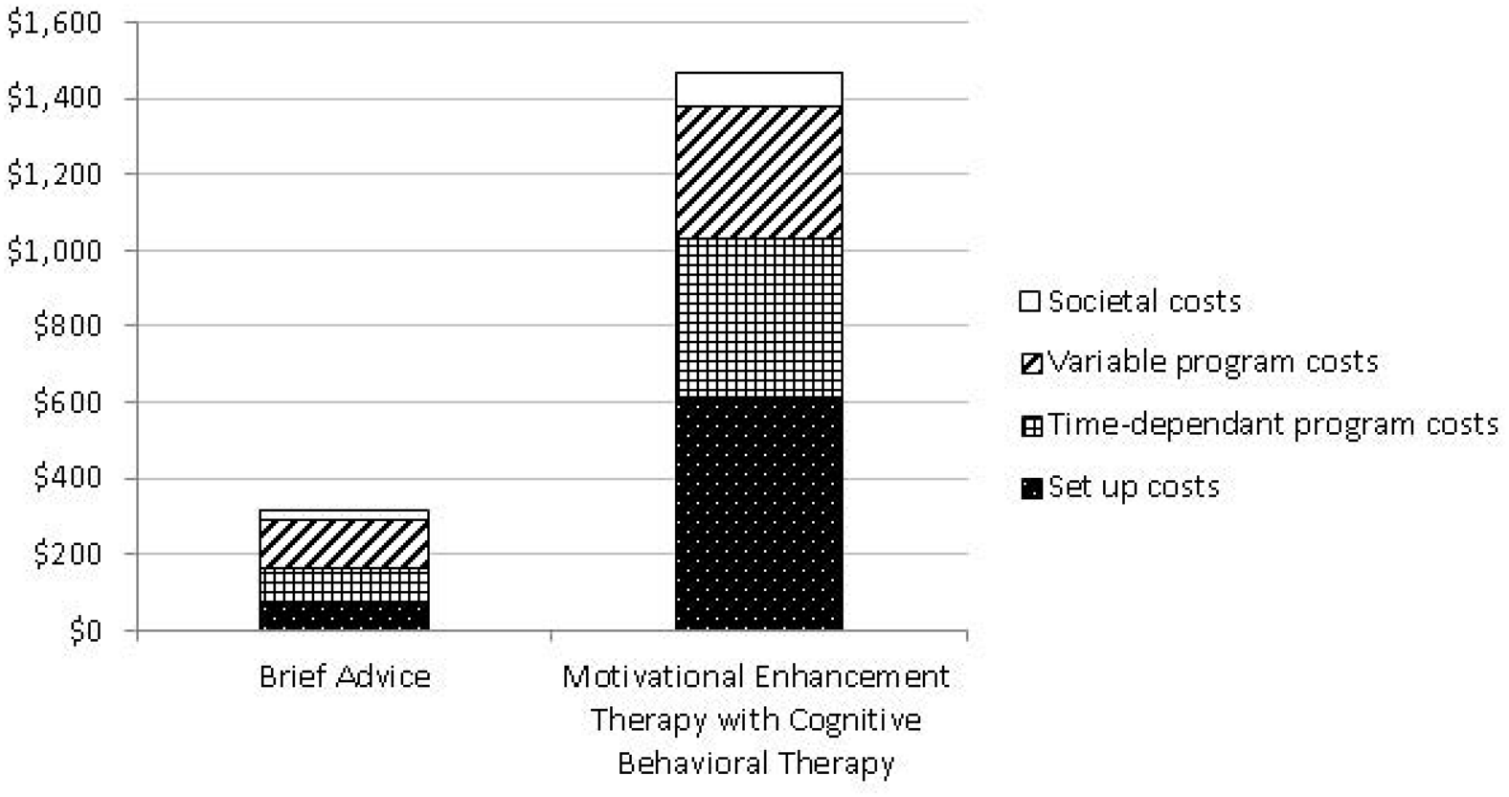
Comparison of per participant cost: Brief Advice and Motivational Enhancement Therapy with Cognitive Behavioral Therapy. This figure illustrates the relative distribution of the four cost categories (i.e., set up costs, time-dependent program costs, variable program costs, and societal costs) for the two intervention arms, i.e., Brief Advice and Motivational Enhancement Therapy with Cognitive Behavioral Therapy, respectively.

Detailed breakdown of the total costs for each intervention was also presented in Table 2, while the relative distribution of the four cost categories (i.e., set up costs, time-dependent program costs, variable program costs, and societal costs) in terms of cost per participant were summarized in Figure 1.

#### Set up costs

Set up costs comprised the largest share (41.75%) of the total costs for the MET-CBT intervention due to the high costs of designing the intervention materials and provider training. It resulted in a total cost of $50,297 or $613 per participant. In contrast, set up costs only accounted for 23.80% of the total costs for the BA intervention ($6,473 total and $75 per participant) due to much fewer resources needed for program designing and training.

#### Time-dependent program costs

Time-dependent program costs made up 28.77% and 28.27% of the total costs for the MET-CBT and the BA interventions, respectively, amounting to $423 and $89 per participant, respectively. Most of these costs were personnel time spent on continued clinic integration including updating clinical documents (e.g., referral information sheet), meetings and other interactions with the clinic to maintain engagement of clinic staff and providers, and resolving any issues about running the program in the clinical setting, as well as the facility and utilities costs for the program administrative office where the therapists and supervisors were housed. Both interventions required minimal investment in equipment and furniture and per participant costs of office and other miscellaneous supplies were quite low.

#### Variable program costs

Variable program costs totaled $345 per participant for MET-CBT intervention compared with $125 per participant for BA, a difference of $220 (Table 2). It comprised the largest component of total costs (39.67%) for BA. The most expensive cost categories were personnel time (include time spent delivering the sessions, screening patients, scheduling visits, and performing urine/breath tests) and urine and breath test supplies. Costs of other intervention materials (e.g., program brochure, worksheets, and information sheets) were modest.

#### Societal costs

Societal costs were comprised of participants’ time in sessions and in travel. As shown in Table 2, the cost of participants’ additional time spent in MET-CBT (totaled $88 per participant) was considerably greater than that spent in BA (totaled $26 per participant). This difference was mostly driven by the longer duration of sessions in the MET-CBT intervention. Societal costs, however, accounted for a relatively small portion of the total costs for both interventions.

### Sensitivity Analyses

We conducted sensitivity analyses to examine the variation in our total cost estimates as a result of variation in key parameters. We were most interested in examining the effects of uncertainties in several areas: number of intervention participants, need for provider training, type of providers delivering the intervention, and participant travel. As shown in Table 3, increasing the number of intervention participants by 25% would considerably reduce the total costs to $1,255 per participant for MET-CBT and $282 per participant for BA. If the program could identify providers who were already capable of delivering the therapy without the need to train them (holding the wage rates constant), per participant costs would be substantially reduced as well ($1,153 per participant for MET-CBT and $285 per participant for BA). If social workers could be trained to deliver the interventions (instead of engaging nurses whose labor was more costly), the program costs would be somewhat reduced for both MET-CBT and BA. In contrast, if the program were replicated in a non-academic setting (i.e., replace resident physicians, interns and fellows with attending physicians), the program costs would become slightly higher. Mode of transportation used by participants (i.e., public transportation versus driving) only minimally affected the total costs of the two interventions, because the interventions were largely integrated into routine prenatal and postpartum visits which reduced the need for separate visit by participants. Although driving resulted in higher transportation expense, the shorter travel time reduced participants’ productivity loss. The overall per participant cost when driving was slightly lower than using public transportation.

**Table 3:** Estimated per participant cost in sensitivity analysis.

Uncertainty Examined	Cost perParticipant	
	Brief Advice(BA)	Motivational Enhancement Therapy with Cognitive Behavioral Therapy (MET-CBT)
**Base Case Analysis**	$316.27	$1,469.31
**One-Way Sensitivity Analysis**		
Increase number of intervention participants by 25%	$282.30	$1,255.49
Exclude training costs^a^	$285.47	$1,152.79
Replace registered nurses (RN) with social workers as MET-CBT therapists	$311.66	$1,350.65
Assume the program was implemented in a non-academic setting^b^	$334.82	$1,470.56
Use driving as mode of transportation for participants	$312.96	$1,455.96
**Two-Way Sensitivity Analysis**		
Increase number of intervention participants by 25% AND exclude training costs^a^	$257.67	$1,002.27
Increase number of intervention participants by 25% AND replace RN with social workers as MET-CBT therapists	$278.61	$1,150.59

a Assume staff members are already capable of running program and delivering therapy.

b Wage rate of resident physicians and fellows were replaced by wage rate of attending physicians.

Finally, when we simultaneously increased the number of intervention participants by 25% and excluded training costs, the expected program costs were substantially reduced to $1,002 per participant for MET-CBT and $258 per participant for BA. Likewise, simultaneously increasing the number of intervention participants by 25% and replacing nurses with social workers as MET-CBT therapists resulted in a per participant cost of $1,151 for MET-CBT and $279 for BA.

## Discussion

While evidence on the efficacy of behavioral interventions to promote drug abstinence in pregnancy and immediately after delivery continues to emerge, there is a dearth of rigorous economic evaluations of these programs. Using data prospectively collected alongside an RCT, we conducted the first micro-costing analysis of an innovative behavioral approach that integrated MET with CBT, as compared to standard BA, within prenatal care for reduction of substance use and HIV risk behavior and promotion of birth outcomes. The MET-CBT program was estimated to cost $1,469 per participant from a societal perspective (versus $316 per participant for the BA program).

Methodologically, consistent with recommendations by the U.S. Panel on Cost Effectiveness in Health and Medicine [13], we applied a micro-costing framework and quality standards developed in previous studies by members of our research team and extended it through operationalizing the costing techniques in the current RCT. This study, along with others we have conducted [23], [24], [25], helped exemplify the execution of micro-costing analysis for health care interventions. This addresses a gap in the literature where in spite of the Panel’s recommendation of micro-costing, existing guidelines for economic evaluations do not provide sufficient specifics for the exact costing methods [16]. In contrast, none of the previous economic evaluations of substance use treatment programs for pregnant women have used a micro-costing method. These studies differed greatly in the approach and evaluation methods used (e.g., direct comparisons of costs versus multivariable regressions, application of per diem costs versus Diagnosis Related Group (DRG)-specific costs) and often did not use standardized cost components because various analysis perspectives have been adopted (e.g., patient, payer, taxpayer), hindering our ability to compare intervention costs between studies [12].

Substantively, we expanded the current literature on substance use treatment for pregnant women by quantifying the economic impact of a behavioral intervention. Prior research in this population has predominantly focused on integrated treatment programs (e.g., multidisciplinary residential care followed by intensive outpatient treatment) or pharmacotherapy (e.g., methadone or detoxification) [34], [35], [36], [37], [38]. Per patient cost of substance use treatment in these programs ranged from $2,535 for detoxification only programs to $10,187 for residential plus outpatient treatment [34], [36], [38], [39]. Compared to these interventions, the behavioral therapy evaluated in our study had a much lower cost per participant, i.e., $1,469. This is consistent with results from prior micro-costing analyses of education and behavioral interventions in other substance use populations (e.g., university and middle school students, emergency department patients), which have generally shown low program costs [23], [40], [41], [42], [43]. Our estimates also compare favorably to findings from a recent micro-costing study on opioid dependence treatment in Malaysia combining naltrexone and buprenorphine with manual-guided counseling [24].

As expected, personnel costs (nurse therapists and obstetric providers) for delivering the intervention sessions and supervising the program comprised the largest share of the MET-CBT intervention costs. Program set up costs, especially intervention material design and training costs, also contributed substantially to the overall cost. Because these set up costs are fixed costs for the program which will be diluted as additional patients are cared for in the program, we anticipate much lower per participant costs for the MET-CBT intervention in the long run. This is supported by our sensitivity analysis where the total cost reduced considerably from $1,469 per participant to $1,153 per participant when training costs were excluded.

The modest cost of the MET-CBT intervention, in conjunction with its potential benefit to reduce maternal and neonatal complications and improve maternal substance use and sexual behavior, suggests potential for cost effectiveness and/or cost saving. Although clinical outcomes from the PRIDE trial showed no significant difference in abstinence rates or days of substance use between the MET-CBT and BA groups up to 3 months post-delivery, there was a trend for reduced substance use among women with a diagnosis of abuse or dependence and a trend for lower preterm birth rate for MET-CBT participants than BA participants [21]. Our current analysis focused on assessing the costs of the MET-CBT and BA interventions. Future cost effectiveness or cost utility analyses integrating cost assessments with clinical outcomes, particularly pregnancy outcomes and longer term substance use and health outcomes for subgroups of women with more serious substance use problems, would enable a more comprehensive understanding of the role of behavioral interventions in improving the care of substance abusing pregnant women.

Several limitations of the study should be acknowledged. First, because our analysis was conducted at two reproductive health clinics located in a single state, the findings may not be generalizable to clinics in other parts of the country. Because wage rate and prices are generally high in the state of Connecticut where the study was conducted [33], [44], we expect the average per participant cost to be lower if the intervention is replicated elsewhere. However, this would also depend on the exact care setting where the intervention is provided. Second, we performed the micro-costing analysis of the MET-CBT program in a research setting. While we took great effort to exclude research-induced costs, this could result in over- or under-estimated costs in some categories (e.g., screening cost, set up of program administrative office) due to challenges of disentangling resource use for clinical versus research programs. Third, our measurement of provider and personnel time costs heavily relied on self-observation by providers and study personnel. While this approach minimized burden of data collection as compared to alternative methods, e.g., direct observation by a trained observer or patient flow analysis [45], it is susceptible to recall bias. However, as we elicited information from multiple providers and personnel members whenever possible, we expect such potential bias to be small.

Despite these limitations, this study provided much needed data for understanding the levels and types of resources necessary for delivering a behavioral intervention integrating MET with CBT for pregnant substance users. Drawing on detailed utilization and unit cost data, the micro-costing analysis offered an accurate account for the costs of implementing such a program and provided essential input data for future cost effectiveness, cost benefit, and/or cost-minimization analyses of this intervention. By allowing clinicians and policy-makers to accurately understand and estimate costs and weigh them against health benefits, this study can facilitate rational allocation decisions in providing substance use treatment for pregnant women in the long run.

## References

[1] Substance Abuse and Mental Health Services Administration (2012) Results from the 2011 National Survey on Drug Use and Health: Summary of National Findings, NSDUH Series H-44, HHS Publication No. (SMA) 12-4713. Rockville, MD: Substance Abuse and Mental Health Services Administration.

[2] Behnke M, Smith VC (2013) Prenatal substance abuse: short- and long-term effects on the exposed fetus. Pediatrics 131: e1009–1024. 23439891 10.1542/peds.2012-3931 PMC8194464

[3] Rayburn WF (2007) Maternal and fetal effects from substance use. Clin Perinatol 34: 559–571, vi. 10.1016/j.clp.2007.09.001 18063105

[4] Van Dyke RB (2011) Mother-to-child transmission of HIV-1 in the era prior to the availability of combination antiretroviral therapy: the role of drugs of abuse. Life Sci 88: 922–925. 21439978 10.1016/j.lfs.2011.03.006

[5] Rodriguez EM, Mofenson LM, Chang BH, Rich KC, Fowler MG, et al (1996) Association of maternal drug use during pregnancy with maternal HIV culture positivity and perinatal HIV transmission. AIDS 10: 273–282. 8882667 10.1097/00002030-199603000-00006

[6] Grivell R, Dodd J, Robinson J (2009) The prevention and treatment of intrauterine growth restriction. Best Pract Res Clin Obstet Gynaecol 23: 795–807. 19615946 10.1016/j.bpobgyn.2009.06.004

[7] Schulte J, Dominguez K, Sukalac T, Bohannon B, Fowler MG (2007) Declines in low birth weight and preterm birth among infants who were born to HIV-infected women during an era of increased use of maternal antiretroviral drugs: Pediatric Spectrum of HIV Disease, 1989–2004. Pediatrics 119: e900–906. 17353299 10.1542/peds.2006-1123

[8] Phibbs CS, Bateman DA, Schwartz RM (1991) The neonatal costs of maternal cocaine use. JAMA 266: 1521–1526. 1880883

[9] Burstyn I, Kapur N, Cherry NM (2010) Substance use of pregnant women and early neonatal morbidity: where to focus intervention? Can J Public Health 101: 149–153. 20524381 10.1007/BF03404362 PMC6974219

[10] Office of National Drug Control Policy (2004) The Economic Costs of Drug Abuse in the United States, 1992–2002. Washington, DC: Executive Office of the President. Publication No.207303.

[11] Taillac C, Goler N, Armstrong MA, Haley K, Osejo V (2007) Early start: an integrated model of substance abuse intervention for pregnant women. Perm J 11: 5–11. 10.7812/tpp/07-013 PMC3057720 21461106

[12] Ruger JP, Lazar CM (2012) Economic evaluation of drug abuse treatment and HIV prevention programs in pregnant women: a systematic review. Addict Behav 37: 1–10. 21962429 10.1016/j.addbeh.2011.07.042 PMC3216632

[13] Gold MR, Siegel JE, Russell LB, Weinstein MC (1996) Cost-effectiveness in Health and Medicine. New York, NY: Oxford University Press.

[14] Tan SS, Rutten FF, van Ineveld BM, Redekop WK, Hakkaart-van Roijen L (2009) Comparing methodologies for the cost estimation of hospital services. Eur J Health Econ 10: 39–45. 18340472 10.1007/s10198-008-0101-x

[15] Heerey A, McGowan B, Ryan M, Barry M (2002) Microcosting versus DRGs in the provision of cost estimates for use in pharmacoeconomic evaluation. Expert Rev Pharmacoecon Outcomes Res 2: 29–33. 19807427 10.1586/14737167.2.1.29

[16] Barnett PG (2009) An improved set of standards for finding cost for cost-effectiveness analysis. Med Care 47: S82–88. 19536018 10.1097/MLR.0b013e31819e1f3f

[17] Frick KD (2009) Microcosting quantity data collection methods. Med Care 47: S76–81. 19536026 10.1097/MLR.0b013e31819bc064 PMC2714580

[18] Chang G, Wilkins-Haug L, Berman S, Goetz MA (1999) The TWEAK: application in a prenatal setting. J Stud Alcohol 60: 306–309. 10371256 10.15288/jsa.1999.60.306

[19] Russell M, Martier SS, Sokol RJ, Mudar P, Jacobson S, et al (1996) Detecting risk drinking during pregnancy: a comparison of four screening questionnaires. Am J Public Health 86: 1435–1439. 8876514 10.2105/ajph.86.10.1435 PMC1380656

[20] Yonkers KA, Gotman N, Kershaw T, Forray A, Howell HB, et al (2010) Screening for prenatal substance use: development of the Substance Use Risk Profile-Pregnancy scale. Obstet Gynecol 116: 827–833. 20859145 10.1097/AOG.0b013e3181ed8290 PMC3103106

[21] Yonkers KA, Forray A, Howell HB, Gotman N, Kershaw T, et al (2012) Motivational enhancement therapy coupled with cognitive behavioral therapy versus brief advice: a randomized trial for treatment of hazardous substance use in pregnancy and after delivery. Gen Hosp Psychiatry 34: 439–449. 22795046 10.1016/j.genhosppsych.2012.06.002 PMC3428516

[22] Yonkers KA, Howell HB, Allen AE, Ball SA, Pantalon MV, et al (2009) A treatment for substance abusing pregnant women. Arch Womens Ment Health 12: 221–227. 19350369 10.1007/s00737-009-0069-2 PMC3103065

[23] Ruger JP, Ben Abdallah A, Cottler L (2010) Costs of HIV prevention among out-of-treatment drug-using women: results of a randomized controlled trial. Public Health Rep 125 Suppl 1 83–94. 20408391 10.1177/00333549101250S111 PMC2788412

[24] Ruger JP, Chawarski M, Mazlan M, Luekens C, Ng N, et al (2012) Costs of addressing heroin addiction in Malaysia and 32 comparable countries worldwide. Health Serv Res 47: 865–887. 22091732 10.1111/j.1475-6773.2011.01335.x PMC3419893

[25] Ruger JP, Emmons KM, Kearney MH, Weinstein MC (2009) Measuring the costs of outreach motivational interviewing for smoking cessation and relapse prevention among low-income pregnant women. BMC Pregnancy Childbirth 9: 46. 19775455 10.1186/1471-2393-9-46 PMC2761847

[26] DeAntonio DA (2010) All-employee hours and earnings for States and metropolitan areas Monthly Labor Review. 133: 41–50.

[27] U.S. Census Bureau Center for Economic Studies. Quarterly Workforce Indicators, 2006–2010. Washington, DC: U.S. Census Bureau Center for Economic Studies. Available: http://lehd.ces.census.gov/applications/qwi_online/. Accessed 2013 April 28.

[28] Internal Review Service IRS Announces 2006 Standard Mileage Rates. Revenue Procedure 2005-78. Available: http://www.irs.gov/uac/IRS-Announces-2006-Standard-Mileage-Rates. Accessed 2013 February 20.

[29] Internal Review Service (Dec. 3, 2009) IRS Announces 2010 Standard Mileage Rates. IR-2009-111. Available: http://www.irs.gov/newsroom/article/0,id=216048,00.html. Accessed 2013 February 20.

[30] Internal Review Service (Nov. 1, 2006) IRS Announces 2007 Standard Mileage Rates. IR-2006-168 IR-2006-168. Available: http://www.irs.gov/uac/IRS-Announces-2007-Standard-Mileage-Rates. Accessed 2013 February 20.

[31] Internal Review Service (Nov. 24, 2008) IRS Announces 2009 Standard Mileage Rates. IR-2008-131 IR-2008-131.Available: http://www.irs.gov/newsroom/article/0,id=200505,00.html. Accessed 2013 February 20.

[32] Internal Review Service (Nov. 27, 2007) IRS Announces 2008 Standard Mileage Rates; Rate for Business Miles Set at 50.5 Cents per Mile. IR-2007-192 IR-2007-192.Available: http://www.irs.gov/uac/IRS-Announces-2008-Standard-Mileage-Rates;-Rate-for-Business-Miles-Set-at-50.5-Cents-per-Mile. Accessed 2013 February 20.

[33] U.S. Bureau of Labor Statistics. Consumer Price Index. Washington, DC. Available: http://www.bls.gov/cpi/. Accessed 2012 June 14.

[34] Daley M, Argeriou M, McCarty D, Callahan JJ Jr, Shepard DS, et al (2000) The costs of crime and the benefits of substance abuse treatment for pregnant women. J Subst Abuse Treat 19: 445–458. 11166509 10.1016/s0740-5472(00)00138-0

[35] Daley M, Argeriou M, McCarty D, Callahan JJ Jr, Shepard DS, et al (2001) The impact of substance abuse treatment modality on birth weight and health care expenditures. J Psychoactive Drugs 33: 57–66. 11333002 10.1080/02791072.2001.10400469

[36] Daley M, Shepard DS, Bury-Maynard D (2005) Changes in quality of life for pregnant women in substance user treatment: developing a quality of life index for the addictions. Subst Use Misuse 40: 375–394. 15776984 10.1081/ja-200030798

[37] Jansson LM, Svikis D, Lee J, Paluzzi P, Rutigliano P, et al (1996) Pregnancy and addiction. A comprehensive care model. J Subst Abuse Treat 13: 321–329. 9076650 10.1016/s0740-5472(96)00070-0

[38] Svikis DS, Golden AS, Huggins GR, Pickens RW, McCaul ME, et al (1997) Cost-effectiveness of treatment for drug-abusing pregnant women. Drug Alcohol Depend 45: 105–113. 9179512 10.1016/s0376-8716(97)01352-5

[39] French MT, McCollister KE, Cacciola J, Durell J, Stephens RL (2002) Benefit-cost analysis of addiction treatment in Arkansas: specialty and standard residential programs for pregnant and parenting women. Subst Abus 23: 31–51. 12444359 10.1080/08897070209511473

[40] Cowell AJ, Brown JM, Mills MJ, Bender RH, Wedehase BJ (2012) Cost-effectiveness analysis of motivational interviewing with feedback to reduce drinking among a sample of college students. J Stud Alcohol Drugs 73: 226–237. 22333330 10.15288/jsad.2012.73.226 PMC3281981

[41] Dunlap LJ, Zarkin GA, Bray JW, Mills M, Kivlahan DR, et al (2010) Revisiting the cost-effectiveness of the COMBINE study for alcohol dependent patients: the patient perspective. Med Care 48: 306–313. 20355261 10.1097/mlr.0b013e3181ca3d40 PMC3140763

[42] Kilmer B, Burgdorf JR, D’Amico EJ, Miles J, Tucker J (2011) Multisite cost analysis of a school-based voluntary alcohol and drug prevention program. J Stud Alcohol Drugs 72: 823–832. 21906509 10.15288/jsad.2011.72.823 PMC3174026

[43] Neighbors CJ, Barnett NP, Rohsenow DJ, Colby SM, Monti PM (2010) Cost-effectiveness of a motivational intervention for alcohol-involved youth in a hospital emergency department. J Stud Alcohol Drugs 71: 384–394. 20409432 10.15288/jsad.2010.71.384 PMC2859787

[44] U.S. Bureau of Labor Statistics. Overview of BLS Wage Data by Area and Occupation. Washington, DC. Last Modified 12 August 2013. Available at: http://www.bls.gov/bls/blswage.htm. Accessed 9 December 2013.

[45] Gorsky RD (1996) A Method to Measure the Costs of Counseling for HIV Prevention. Public Health Reports 111: 115–122. 8862166 PMC1382052

